# Low-Cost Method and Biochip for Measuring the Trans-Epithelial Electrical Resistance (TEER) of Esophageal Epithelium

**DOI:** 10.3390/ma13102354

**Published:** 2020-05-20

**Authors:** Daniel Puiu Poenar, Guang Yang, Wei Keat Wan, Shilun Feng

**Affiliations:** 1 VALENS-Centre for Bio Devices and Signal Analysis, EEE School, Nanyang Technological University, Blk S1, Level B4, Unit 06 (S1-B4b-06), 50 Nanyang Avenue, Singapore 639798, Singapore; YANG0389@e.ntu.edu.sg (G.Y.); shilun.feng@ntu.edu.sg (S.F.); 2 Histopathology Department, Singapore General Hospital (SGH), 20 College Road, Academia, Level 10, Diagnostic Tower, Singapore 169856, Singapore; wan.wei.keat@singhealth.com.sg

**Keywords:** TEER measurement, tissue biochip, tissue viability, esophageal epithelial integrity, Lab-on-a-Chip, Ussing chamber

## Abstract

Trans-epithelial electrical resistance (TEER) is a good indicator of the barrier integrity of epithelial tissues and is often employed in biomedical research as an effective tool to assess ion transport and permeability of tight junctions. The Ussing chamber is the gold standard for measuring TEER of tissue specimens, but it has major drawbacks: it is a macroscopic method that requires a careful and labor intensive sample mounting protocol, allows a very limited viability for the mounted sample, has large parasitic components and low throughput as it cannot perform multiple simultaneous measurements, and this sophisticated and delicate apparatus has a relatively high cost. This paper demonstrates a low-cost home-made “sandwich ring” method which was used to measure the TEER of tissue specimens effectively. This method inspired the subsequent design of a biochip fabricated using standard soft lithography and laser engraving technologies, with which the TEER of pig epithelial tissues was measured. Moreover, it was possible to temporarily preserve the tissue specimens for days in the biochip and monitor the TEER continuously. Tissue responses after exposure tests to media of various pH values were also successfully recorded using the biochip. All these demonstrate that this biochip could be an effective, cheaper, and easier to use Ussing chamber substitute that may have relevant applications in clinical practice.

## 1. Introduction

The measurement of the Trans-Epithelial/Endothelial Electrical Resistance (TEER) is a widely accepted quantitative technique for in vitro assaying the barrier integrity of epithelial/endothelial tissues or monolayer epithelial cell cultures, as well as assessing ion transport and/or the quality (or operation) of the tight junctions. The measurement principle is straightforward; by applying an A.C. electrical signal across electrodes placed on both sides of the barrier, the TEER can be calculated from the applied voltage and resulting current established in the circuit. Higher TEER implies better barrier integrity.

**Figure 1:**
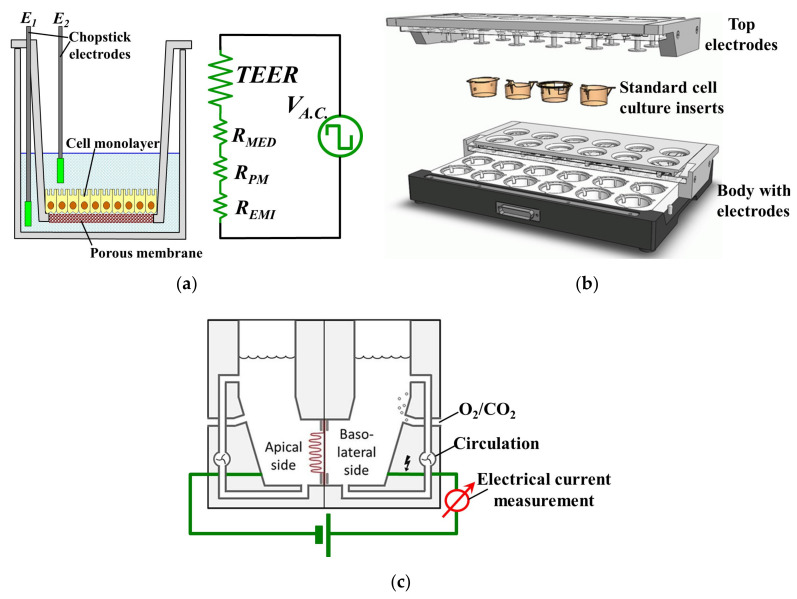
Present-day systems used for tissue/tight junctions’ characterization: (**a**) Standard TEER measurement set-up and its corresponding complete circuit (adapted from [2]. Copyright (2020) with permission from Springer Nature). The ohmic resistance of the cell layer (TEER), the cell culture medium in the upper and lower compartment (*R_MED_*), the membrane of the filter inserts (*R_PM_*) and electrode-medium interface (*R_EMI_*) all contribute to the total measured electric resistance; (**b**) The CellZscope^®^ machine (adapted from [2]. Copyright (2020) with permission from Springer Nature). (**c**) The Ussing chamber system (adapted from [8]. Copyright (2020) with permission from Springer Nature). An A.C. bias may be used instead of the D.C. one shown in the picture, to avoid heating and electrode polarization.

In the case of monolayer cell cultures, measuring TEER is relatively simple: cells are plated and cultured in a Transwell insert whose bottom is a porous membrane permeable to media and ions. A pair of chopstick-shaped electrodes are inserted into the media to measure the TEER, with one leg in the insert and the other leg in the outer well. The interested reader can find more information on this topic in Wegener’s early work [1], or in the reviews of Benson et al. [2] and of Srinivasan et al. [3], respectively. Figure 1a illustrates schematically the set-up, with a cellular monolayer cultured on a semipermeable filter insert that defines a partition for apical and basolateral compartments, each contacted by a chopstick-like electrode [2]. However, the resulting TEER readings obtained with this macroscopic set-up inherently include a few sources of potentially significant errors [2]. Thus, the measured TEER values depend strongly on the shape and position of the probing electrodes. Vibrations easily cause significant disturbances and the reproducibility of electrodes’ placements is also a serious concern, while the inherent inhomogeneity of the electric field across the cell layer typically leads to a systematic overestimation of the measured TEER [2]. A further improvement was provided by the CellZscope^®^ machine (cellZscope, nanoAnalytics GmbH, Muenster, Germany) which eliminated most of the previous drawbacks. It comprises several wells which can all be cultured and measured simultaneously (e.g., in Transwell inserts with 6, 12 or 24 wells), each with its own electrodes that measure the A.C. impedance of barrier-forming cell cultures growing on the permeable membranes of standard cell culture inserts, providing more valuable information than TEER [4]. It is also the only way with which information regarding the evolution of the cell culture can be obtained because light microscopy is no longer feasible for Transwell cultured cells due to the presence of the supports which hinders microscopic observation [5]. The schematic structure of the CellZscope^®^ is shown in Figure 1b. However, this impedance measurement is typically used just to signal when complete confluence of cells in a continuous monolayer was achieved, and thus the cells can be harvested and used for other purposes. Moreover, impedance measurement is more difficult and requires a more complex measurement set-up and data processing in order to derive the relevant parameters of interest from the measurements, after which correctly interpreting these parameters and extracting the right and meaningful conclusions is another non-trivial task.

This explains why TEER measurement is considered the easiest, fastest, and most convenient tool to monitor the quality and behavior of cells cultured on porous supports. Due to its simplicity and usefulness, the TEER method was also integrated with lab-on-a-chip technology to realize promising biodevices [6,7].

Unfortunately, such in vitro tests, although cheap and reasonably easy to do, have serious drawbacks. First, a simple monolayer of cells cannot capture the complex behavior of living tissue, and many cell types are not even identical with those present in the actual organ of interest but relatively similar cell lines are used instead. Second, such measurements are static and can study a limited number of variables and only for a very specific application. Last but not least, they are time-consuming and require a large volume of reagents and culture media.

A much more attractive alternative is to employ instead actual tissues biopsied from the organ of interest. Such tissues are much better biological models than monolayer cell cultures for in vitro experiments because they are more physiologically relevant to the environment *in vivo* that is being studied. However, much less research employed TEER of biopsied tissues due to its challenges. Unlike monolayer cell cultures, measuring the TEER of living tissues using the Transwell insert method is no longer feasible because a tissue specimen simply placed in the Transwell cup will not be adherent to the porous membrane. This means that there will be many gaps between the edge of the specimen and the wall/porous membrane of the Transwell insert. These gaps become channels for fluid and ion transportation which lead to incorrect and unreliable TEER measurements.

The solution for these problems is the Ussing chamber, shown in Figure 1c, which has been used as the gold standard for assessing TEER of tissue specimens [8]. Thus, TEER has been heavily involved in many epithelial tissue-related studies of various topics of interest, such as the permeability of the blood-brain barrier [9,10], the integrity and/or the metabolism of human intestinal mucosa [11], intestinal drug delivery [12], the factors affecting the development of tight junctions [13], the response of tight junctions to proinflammatory cytokines [14], the effect of absorption enhancers on the permeability of intestinal epithelial cells [15] and investigating the pathways of ion transport across epithelial cells [16].

As shown in Figure 1c, an Ussing chamber system generally comprises two half-chambers with a perfusion system, and a piece of epithelial tissue is sandwiched by the two half-chambers in such a way that each side of the tissue is isolated and faces a separate half-chamber. Pathologists and gastroenterologists have been using the Ussing chamber to study the esophageal epithelial integrity in various diseases of the GastroIntestinal Tract (GIT). It was shown that esophageal biopsies had modified TEER values in patients with various GIT affections such as Barrett’s esophagus, gastro-esophageal reflux disease (GERD) [17,18] that also includes the non-erosive reflux disease (NERD) [19,20], celiac disease [21], as well as microscopic colitis (MC), collagenous colitis and treated lymphocytic colitis [22], all indicating disturbances in ion transport and barrier function, with typically a significantly decreased epithelial resistance.

Although the Ussing chamber system proved to be accurate and reliable, it has serious drawbacks. Most importantly, the number of specimens that can be measured at a time is limited by the number of chambers available, which greatly limits the type of experiments can be carried out. Other significant operational limitations include the lack of ability to preserve the specimen for relatively longer periods and difficulty in quickly introducing trace amounts of treatment molecules such as labeling dyes or therapeutic drugs. Finally, it is voluminous, cumbersome to use, and has a relatively high cost.

This paper first reports the development and successful usage of a low-cost home-made “sandwich ring” method for measuring TEER of epithelial tissues. The tissue can be mounted in a specimen holding set which consists of one pair of polydimethylsiloxane (PDMS) rings and one pair of ring magnets. The performance of the “sandwich ring” method was certified by performing TEER measurements on porcine esophageal epithelia. Next, we applied the same underlying idea of this “sandwich ring” method to develop a biochip made of PDMS and polymethyl methacrylate (PMMA) with which real-time continuous TEER measurements of tissue samples were performed. We believe this to be the first biochip being reported enabling both extended viability and TEER continuous measurements of biopsied tissue samples. The outstanding features of this biochip include the ability to allow temporarily culturing of the tissue on-chip, easiness in carrying out chemical treatments, re-usability, low fabrication cost and small size. Such a biochip could thus be used to easily and quickly assess quantitatively the type and severity of various gastro-esophageal deficiencies, to compare similar afflictions between different patients, or estimate the efficiency of treatment by comparing variations in time for the same patient. It will also be shown later in this paper that these TEER variations are directly and strongly correlated with variations in the tissue’s permeability. Both our “sandwich ring” method and the biochip can thus have very important applications in clinical practice with which such valuable assessments can be performed.

## 2. Materials and Methods

PDMS (SYLGARD 184) was purchased from Dow Corning and 1 mm thick PMMA sheets were bought from a local workshop. Small assembly components and tools including ring magnets, stainless steel screws, epoxy adhesives, surgical scissors and scalpel were purchased from RS Components. Fetal Bovine Serum (FBS) and phosphate-buffered saline (PBS) solutions were purchased online from ThermoFisher Scientific Singapore. All other bioreagents/chemicals such as Keratinocyte Growth Medium (KGM), Leibovitz’s L-15 medium, fluorescein isothiocyanate–dextran (FITC-Dextran), sodium alginate, acetic acid, dispase II enzyme, 0.25% trypsin-ethylenediaminetetraacetic acid (EDTA) and antibiotic antimycotic (100×) solutions were purchased from Sigma Aldrich Singapore (Sigma-Aldrich Pte Ltd, 2 Science Park Drive, #05-01/12 Ascent Building, Singapore 118222). Fresh porcine esophagus was harvested from pigs euthanized in Singapore General Hospital (SGH) and delivered to the lab for subsequent processing within three hours. During transportation, the harvested esophagus was kept in ice-cold Leibovitz’s L-15 medium.

### 2.1. Peeling Off the Mucosal/Epithelium Layer from the Porcine Esophagus

Prior to processing the esophagus sample, all surgical tools were rinsed with 70% ethanol followed by UV exposure for 1 h for sterilization and all the processing procedures were conducted in a biosafety cabinet. First the esophagus was cut open with a pair of surgical scissors and the muscular layer was stripped off from the mucosa and submucosa layer using tweezers. This reduced the thickness of sample, from an initial value of about 4 mm down to ~1.5 mm. Next, the harvested tissue was divided into shorter segments (about 5 cm long) using a scalpel. These tissue segments were subsequently washed with PBS solution three or four times until the solution remained clear. Then each segment was further cut into smaller pieces of 2 cm × 2 cm and immersed in 0.25% trypsin-EDTA solution for 5 min, followed by thorough rinsing with PBS. This latter step is necessary to remove blood cells as well as connective tissue cells present in the sample [23]. After the trypsin treatment, the samples were kept in L-15 medium supplemented with 10 U/mL dispase II enzyme at 4 °C for 6 h in order to remove the bonding between the mucosal and subcostal layer without harming the integrity of the mucosal layer. Finally, the mucosal layer was carefully peeled off from the submucosa. This method was reported by Compton et al. [24]. The harvested mucosal/epithelial samples, with a thickness between 400–600 µm, were either used immediately for measurements or temporarily maintained (in KGM culture media supplemented with antibiotic antimycotic solution) for later usage in an incubator at 37 °C with an ambient containing 5% CO_2_. The thickness of the completely dried epithelial tissues was measured with a vernier caliper to be between 150–200 µm.

### 2.2. Fabrication of the “Sandwich Ring” and Biochip

All the PDMS components were fabricated using soft lithography, with molds 3D printed in polylactic acid (PLA) with a 3D printer model Ultimaker 3. Liquid PDMS was mixed with curing agent in a ratio of 10:1 and the mixture was poured into the mold, degassed in a vacuum desiccator for 1 h and cured in an oven at 60 °C for 6 to 8 h. The cured PDMS was then carefully peeled off from the mold using tweezers. One of the major issues of the 3D printed molds is that the mold surface is not perfectly smooth, leading to a relatively rough PMDS surface. These imperfections may prevent tight sealing with other surfaces and would thus result in leakages. This problem was addressed by a simple spin-coating technique. Basically, a thin layer of liquid PDMS was first created on a clean Si wafer 4 inch in diameter using a spin coater at 2000 rpm. The PDMS components were placed on the wafer with the non-smooth surface facing downwards to allow liquid PDMS to stick onto the non-smooth surface. Then the PDMS components were transferred onto another clean wafer, followed by degassing in a vacuum desiccator for 30 min. Lastly, the wafer with PDMS components was placed on a hot plate at 90 °C for 30 min. Once cured, the PDMS components can be peeled off from the wafer and the smoothness of the surface is greatly enhanced.

The PMMA perfusion channels which were used to assemble the biochip were fabricated by using a CO_2_-laser (Universal Laser Systems M-360) to engrave a piece of PMMA sheet in order to realize the channel patterns. The laser power and scanning speed were first tested to make sure they were adequate the cut through the PMMA sheet. The optimal cutting recipe eventually adopted to cut two PMMA layers employed 70% laser power at 4% scanning speed. One of the PMMA layers contained the two-side open channel while the other PMMA layer serves as seal layer with some small holes as fluid inlet/outlet and for electrodes insertion. After placing the two layers together, the top and bottom fluidic channels were realized [25]. The two PMMA layers were thermally bonded to prevent any fluid leakage. For this purpose, the two layers were clamped tightly and maintained in an oven at 95 °C for 20 min. [26]. The Ag/AgCl electrodes used in the biochip were prepared by immersing sanded Ag wires of 0.3 mm diameter into chlorine bleach until the Ag wire turned black.

Both the PDMS rings used in the “sandwich ring” as well as the biochip can be re-used, if so desired. In such a case, every time before loading a new piece of tissue sample, all the biochip components (or the PDMS rings) are immersed in 70% ethanol for at least 15 min., then dried in a vacuum desiccator for one hour, followed by exposure to UV light in a biosafety cabinet for 1 h to achieve sterilization. Once all these operations were performed, the tissue sample was cut into 6 mm disks with a biopsy punch and loaded either between the rings (for the “sandwich ring”), or into the central groove between the central PDMS layers of the biochip. At last, the four PMMA layers and the two PDMS layers of the biochip are assembled together and secured with screws.

### 2.3. TEER Measurements Using the “Sandwich Ring” Tissue Holder

A piece of an epithelium sample (that had initially been prepared as mentioned above) was placed on a piece of membrane filter (Whatman^TM^, 0.2 µm pore size) wetted with PBS. Next, disk-shaped specimens were prepared by cutting the epithelium sample using a disposable 6 mm biopsy punch. This disk specimen would fit exactly in the central groove of a PDMS ring. Then the other PDMS ring was placed above it to sandwich the specimen, and a pair of ring magnets purchased from Eclipse Magnetics Singapore (75 Rowell Road, Singapore 208011; product no.: N832RS; outer and inner hole diameters: 15.4 mm and 3.3 mm, respectively; 3.25 mm thick; pull force: 3.3 kg) were used to sandwich in-between them the PDMS rings, thus forming the complete specimen holder.

The TEER measurements were carried out using a voltohmmeter (EVOM2, World Precision Instruments) in a TEER chamber (Endohm-6, World Precision Instruments). Prior to measurement, 100 µL of KGM medium were added in the Endohm-6 chamber to wet the bottom surface. The bottom space of the specimen holder was filled with 60–70 µL medium and the specimen holder was mounted in Endohm-6. Then the top space of the Endohm’s specimen holder above the tissue was filled with 100 µL medium. Filling up the spaces of the specimen holder that are above and below the tissue with KGM culture media with a conductivity of ~1.4 S/m ensures a direct low resistance electrical connection between the electrodes and the tissue surface. The Endohm-6 specimen holder was then closed with its cap and the position of the electrode on the cap was carefully adjusted to make sure it was touching the medium in the top space. This ensured that an electrical circuit was formed between the two pairs of electrodes through the tissue sample, and TEER values could be measured and recorded after connecting the Endohm-6 chamber to EVOM-2.

The accuracy of the “sandwich ring” method was verified by measuring the TEER values of monolayer cell cultures and then compared with those obtained from the standard method (Transwell). In brief, Caco-2 cells (a colon epithelial cell line) were plated on the porous membranes of 4 inserts of Transwell dishes and cultured until full confluence. Then the TEER of these monolayer cell cultures was measured with a pair of chopstick electrodes and our EVOM2 voltohmmeter. The measured value was 1154  ±  97 Ω·cm^2^. Subsequently, these porous membranes with cells were carefully cut off from the Transwell inserts and their TEER was measured again using our “sandwich ring” method. The measured TEER was 1071  ±  65 Ω·cm^2^. The *p*-value for the two data sets is 0.161, indicating that the difference between these two values is statistically insignificant. Hence, the accuracy of our “sandwich ring” method was confirmed.

When sufficient data points could be collected for our measurements, the graphs illustrating the observed variations as a function of the modified parameters included both the data points and the least squares fitting curves which could be found for those data points. This is because such fitting curves would more clearly illustrate the overall variation trend, unlike a jagged multi-segmented line resulted from point-to-point connection that is less accurate and more prone to errors given the inherent spread in the statistical set of collected data. For brevity, the values of the coefficients of the fitting equations and of the measured data points are not mentioned here but are included in the Supplementary Materials file.

Each data point in all the graphs presented in the paper is the mean value resulted from several separate measurements. The specific number *n* of measured values whose average provided each of the plotted data points is clearly specified in the caption of that respective Figure, and the accompanying error bars represent the standard deviation σ.

### 2.4. Preserving Tissue Specimens in the Biochips and Performing TEER Measurements

Prior to specimen mounting, all biochip components were rinsed with 70% ethanol followed by UV exposure for 1 h for sterilization. A 6 mm disk specimen was placed in the groove of one PDMS layer of the biochip and was then covered with the other PDMS layer to sandwich the specimen. Then the PDMS layers with the specimen were sandwiched themselves between the top and bottom PMMA channels which were stacked together and secured tightly with screws. The two perfusion flows were supplied using a peristaltic pump at a flow rate of 0.6 mL/h from a KGM medium reservoir which was maintained in an incubator at 37 °C and 5% CO_2_. To perform the TEER measurements, the four electrodes were connected to the EVOM2 using alligator clips.

Any outlier TEER readings (less than 100 or more than 700 Ω·cm^2^) obtained with the biochip were discarded. The low readings were probably caused by leakages in the experimental setup while the excessively high readings were most likely due to air bubbles trapped in the central hole.

### 2.5. Fluorescence Intensity Measurements to Determine the Tissue Permeability

The fluorescence intensity was measured using a plate reader (Tecan Infinite 200 Pro) with the software i-control™. The experimental procedure was as follows. Medium with added 5 μg/mL FITC-dextran was perfused into the upper (apical) channel of the biochip and the effluent from the lower (basal) channel was collected. Specifically, 50 µl of the same sample solution were added to each of the 4 wells out of the total of 8 wells of the same column of a 96-well (12 columns × 8 rows) Transwell plate, and each column held liquid from different samples. The Transwell plate was then inserted in the plate reader and the concentration of the FITC-dextran in the bottom effluent was quantified by measuring the fluorescence intensity at 530 nm, for an excitation wavelength of 480 nm. An increase in the FITC-dextran concentration in the lower channel effluent would imply an increased tissue permeability.

### 2.6. Tissue Sectioning and Haematoxylin & Eosin Staining for Histological Analysis

The tissue specimens were first fixed in a solution of 4% formalin, followed by dehydration with escalated concentrations of ethanol from 20% to 99%. Then the sample was embedded in paraffin blocks, followed by being sectioned vertically into slices 4 µm thick using a manual microtome (Leica RM2235) to reveal the cross-section of the specimens. The sliced samples were placed on glass slides and stained with haematoxylin and eosin in a fully automated stainer (Leica CV5030). Lastly, the stained samples were analyzed using a light microscope (Olympus BX43).

## 3. Results

### 3.1. Design and Fabrication of the “Sandwich Ring”

**Figure 2:**
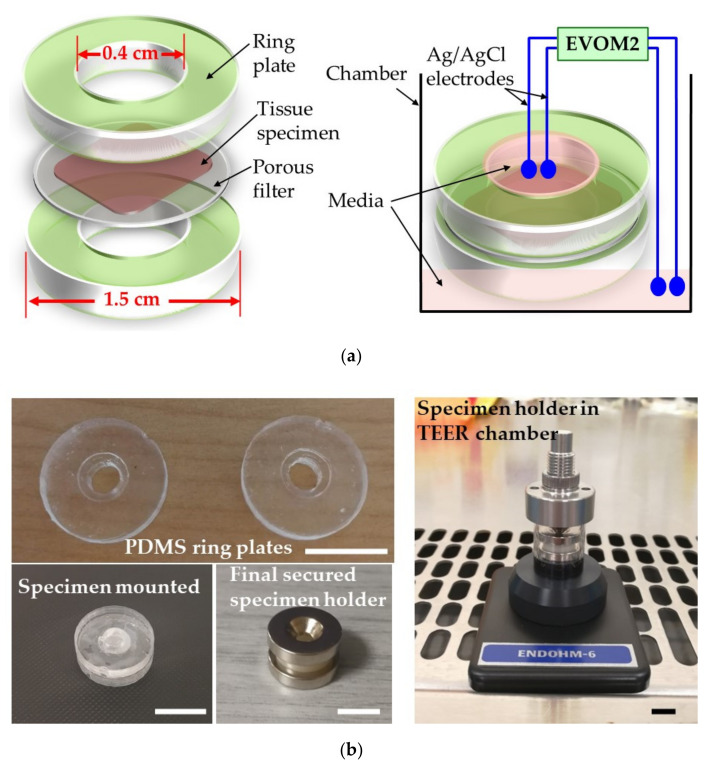
Design concept and experimental setup of the “sandwich ring”: (**a**) The drawing on the left side is a schematic representation of an exploded view of the “sandwich ring”, clearly illustrating how the tissue is clamped in-between two PDMS rings with a central hole smaller than the size of the specimen. For simplicity, the central grooves of the rings are not indicated in the drawing. The drawing on the right side shows in a simplified manner the insertion and usage of the “sandwich ring” in the Endohm-6 TEER chamber. (**b**) Images of the as-fabricated PDMS rings and the experimental setup for specimen loading and TEER measurement. The disc-shaped specimen is mounted in the central groove of the rings and secured by a pair of ring magnets. This secured specimen holder is then placed in an Endohm-6 TEER chamber with one pair of electrodes at the bottom and the other at the cap. The scale bars in all photos represent 1 cm.

The key to success in performing reliable TEER measurements of a tissue sample is to secure the specimen by tightly clamping it in such a way that no fluid leakage occurs between the apical and basal sides, which would thus be completely electrically isolated from one another. Being inspired by the concept underlying the Ussing chamber, the “sandwich ring” method was designed and developed to achieve this purpose, as illustrated in Figure 2a. The ring plates are made of PDMS by soft lithography with a 3D-printed mold. The size of the ring is 15 mm in diameter and 2 mm thick, with a central concentric groove 6 mm in diameter and 0.3 mm deep, and a central hole of 4 mm in diameter. The tissue sample was prepared and mounted between the PDMS rings and neodymium magnets to form the complete specimen holder as described previously in Section 2.3. Inside it, the tissue was firmly pressed between the PDMS rings with a central hole with a size smaller than the tissue specimen to allow contact with the electrodes via the highly conductive top and bottom media. The fact that the PDMS rings were forcefully and tightly clamped together by the over- and underlying ring magnets prevented any leakage around the periphery of the tissue sample and effectively isolated the apical and basolateral sides of the tissue from one another other, similar to what is achieved in an Ussing chamber.

The specimen holder was placed in the ENDOHM-6 chamber and contacted using two pairs of electrodes connected to the commercial EVOM-2 voltohmmeter, as was also described in Section 2.3, after which TEER measurements were performed.

Figure 2b shows the as-fabricated ring plates and the experimental setup for specimen loading and TEER measurement.

### 3.2. TEER Measurement Results Obtained Using the “Sandwich Ring”

**Figure 3:**
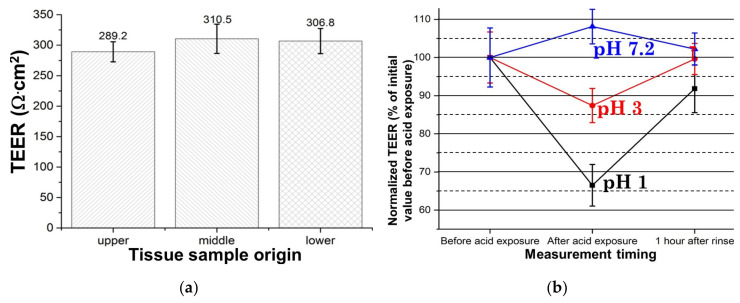
TEER measured values using the “sandwich ring” method: (**a**) TEER values from upper, middle, and lower thoracic porcine esophagus, respectively. The *p*-value = 0.0954 (*n* ≥ 5) for our obtained data set shows that no statistically significant difference exists between the results for the samples from the upper and middle esophagus; (**b**) TEER changes in the tissue specimens exposed for 20 min. to environments of various pH values and after a 1 h recovery (*n* = 4). Irreversible damage was induced on the tissue treated with the pH 1 medium. (The numerical values for the data shown in Figure 3a,b can be found in Table S10 in the Supplementary Materials).

Epithelial tissues from three different segments of porcine esophagus, namely from upper, middle, and lower thoracic esophagus, respectively, were measured using the “sandwich ring” setup. The TEER values of these three segments from the same batch of porcine esophagus are plotted in Figure 3a. It should be noted that the immediate reading from the EVOM2 voltohmmeter is electrical resistance measured in ohms [Ω]. Since this electrical resistance is dependent on the cross-sectional area of the specimen, a unit of [Ω·cm^2^] is commonly used instead for TEER results. This unit of [Ω·cm^2^] is equivalent to resistivity × tissue thickness, hence it is independent of the exact size of the measured tissue. To express the results in this new unit, the measured electrical resistance values were multiplied with the surface area of the central hole of the specimen holder.

The final results shown in Figure 3a demonstrate that there is no significant difference in the TEER values from the three segments, indicating uniformity in the same esophagus. Although the TEER of the upper thoracic esophagus is 289.2 ± 16.5 Ω·cm^2^ and that of the lower thoracic esophagus is 310.5 ± 24 Ω·cm^2^, the *p*-value for our data set is 0.0954, indicating that the difference between these two values is statistically insignificant. These results agree well with those of Woodland et al. who also found no differences between the TEER values of human proximal and distal esophageal mucosa [27]. Nevertheless, it is worth mentioning that the TEER values of porcine esophagus tissue samples could vary from batch to batch. The average measured TEER values ranged from 274.1 to 350.5 Ω across the 6 batches of esophagus epithelial tissue that we measured. This is probably due to the variations in the quality of the harvested esophagus. However, it is difficult to make a direct comparison with other reported TEER values measured using an Ussing chamber, because there is very little previous work on porcine esophagus, but Zhang et al. recently reported a measured value of 110 ± 7 Ω·cm^2^ for esophageal epithelium (of unspecified thickness) from guinea pigs [28]. This mean value is smaller than ours but large variations exist between TEER values reported by various groups studying epithelial integrity. For instance, in one study using human endoscopic esophagus epithelial biopsies from 15 healthy volunteers, the measured TEER values ranged from 106 to 368 Ω·cm^2^ with a mean value of 196.9 ± 16.3 Ω·cm^2^ [18]. Another work reported values between 241 to 324 Ω·cm^2^ with a mean value of ~282.5 Ω·cm^2^ for a batch of human endoscopic biopsies of esophageal epithelium from 13 normal volunteers [29], while another group reported values between 80 to 133 Ω·cm^2^ with an almost halved mean value of 107 Ω·cm^2^ for human endoscopic biopsies of esophageal epithelium from 25 control subjects [30].

The first reason for these differences is most probably related to technical issues, such as variations in experimental setup [31]. Indeed, the review article by Srinivasan et al. outlines that using different measuring equipment may lead to very different TEER measured values [3]. The summary table presented in that review paper indicates that the TEER of a CACO-2 cell culture was between 1400–2400 Ω when using an EVOM voltohmmeter, while a value of only 250 Ω was measured using a Millicell-ERS (Electrical Resistance System) system. Additionally, the sample thickness is also another crucial factor because sizable variations in the tissue thickness will determine nonuniformities of the current density through the sample. Such nonuniformities were also indicated as one of the key reasons for erratic TEER values [3]. Therefore, we suggest that the discrepancy between our measured TEER and other reported values, as well as the large variation between the values reported in the literature, are due to two essential factors: the different measurement setup and equipment adopted in each case, and the significant differences between the thicknesses of the samples used by different researchers (which were not specified in any of the papers cited above), even within the same batch.

In a healthy esophagus, the epithelium of its mucosa serves as a barrier against any luminal reflux. In patients with erosive esophagitis, this barrier function is visibly impaired and the gastric reflux can easily reach the exposed areas to cause mucosal excitation which leads to heartburn. However, in patients with NERD, the esophageal mucosa appears normal on endoscopy and the mechanisms leading to heartburn are less clear [32]. Hence, many researchers have been using TEER to compare the response of both diseased and healthy esophagus to acid [33] and ionic stimulations [34], in an attempt to better understand the pathology of esophageal diseases. Given the importance of such studies, we considered it highly relevant to demonstrate the feasibility of our “sandwich ring” setup for performing such acid exposure tests. Briefly, fresh epithelial specimens were immersed in acidified KGM media for 20 min. Specifically, pH 1 and the pH 3 solutions were prepared by supplementing the base KGM medium with hydrochloric acid and acetic acid, respectively. After TEER measurements, the acid treated specimens were thoroughly rinsed with normal media and incubated at 37 °C for 1 h to recover. Next, new TEER measurements of the recovered specimens were performed. The results are normalized as a percentage of the initial TEER value before acid exposure and plotted in Figure 3b. It is observed that TEER dropped by 33.5% for the sample exposed to the pH 1 medium, but only with 12.6% for exposure to the pH 3 medium. Additionally, after rinse and the incubation period, the tissues treated with pH 3 media almost fully recovered. In contrast, the samples exposed to pH 1 medium restored to only 91.8% of their original TEER value, indicating that some irreversible damages had been induced during the acid treatment. In contrast, TEER increased by 8.1% in the control group in which the sample was exposed to a PBS solution with pH 7.2. After rinse, it reached 102.2% of the initial value.

Similar results had been reported in the previously cited work of Zhang et al., who noted a variation of the mean TEER value from 110 ± 7 Ω·cm^2^ for normal esophageal epithelium of guinea pigs to 92 ± 20 Ω·cm^2^ for samples that had been exposed to HCl in Krebs bicarbonate solution (KBS) with a pH of 2.1 for 30–120 min., followed by rinsing with fresh KBS (pH 7.4) for 30 min. [28]. Likewise, Rinsma et al. exposed endoscopic human esophageal biopsies to an acid solution of pH 1 for 30 min., resulting in a decrease of the TEER values by 52 ± 5%, but which recovered to about 90% of its initial value 2 h after exposure [20]. Takezono et al. had carried out acid exposure tests on monolayer gastric epithelial cell cultures and their results showed that the TEER gradually decreased to about 80% after a 60 min. exposure to a pH 3 solution, while those exposed to a control medium (pH 7.4) had only a transient decrease in the first 10 min. after which they recovered to pretreatment values and remained constant for at least 60 min. [35].

### 3.3. Design and Fabrication of a Tissue Biochip

Once the feasibility and usefulness of the “sandwich ring” method was demonstrated, we moved one step further and applied the same concept for the design and development of a tissue biochip. This biochip was intended to have a dual function, i.e., to enable both temporarily preserving the tissue, and thus extending its viability, as well as performing TEER measurements.

**Figure 4:**
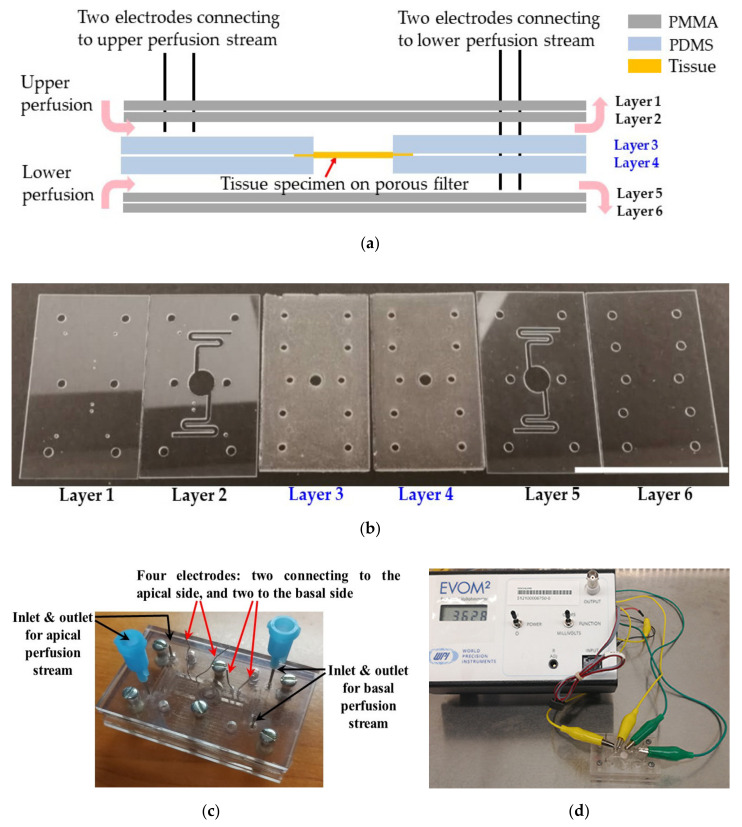
Design concept and experimental setup of our dual-function tissue biochip: (**a**) Cross-sectional view of the biochip structure; (**b**) Image of all the six component layers that constitute the biochip. Layers 1 and 2 provide the upper perfusion channel; Layers 3 and 4 form the tissue loading layers, and Layer 5 and 6 make up the lower perfusion channel. The scale bar represents 5 cm. (**c**) Final assembled biochip (5.5 cm × 3.5 cm). Two perfusion flows were supplied to preserve the tissue specimen in the chip using a peristaltic pump at flow rate of 0.6 mL/h from a KGM medium reservoir maintained in an incubator at 37 °C and 5% CO_2_. (**d**) TEER measuring setup.

By integrating the strengths of TEER and lab-on-a-chip technology, the biochip can monitor the tissue’s TEER continuously for several days. Figure 4a illustrates the structure of the tissue biochip. The design idea of our tissue biochip originates from the “sandwich ring” method and previously reported biochips designed for monolayer cell culturing and TEER measurement [36,37]. This design comprises two PDMS layers—each 2 mm thick—with a 6 mm circular groove (indentation) and a central hole 4 mm in diameter. The PDMS layers were fabricated using soft lithography with a 3D printed mold. The upper and lower perfusion channels were fabricated from PMMA sheets engraved by using a CO_2_ laser. Four Ag/AgCl wires were inserted into the biochip to connect the upper and lower perfusion, functioning as electrodes for TEER measurement. All the six component layers that constitute the biochip are shown in Figure 4b. The first two PMMA layers at the left (layer 1 and layer 2) constitute the upper perfusion channel. After thermally bonding these two layers together, a one-sided open channel is achieved. The last two PMMA layers on the right (layer 5 and layer 6) are used in the same way to make up the lower perfusion channel. The two PDMS layers in the middle (layer 3 and layer 4) serve as specimen holder. The layers have dimensions of 5.5 cm × 3.5 cm and the thicknesses of the PMMA and PDMS layers are 1 mm and 2 mm, respectively. The six layers are assembled and secured with screws to realize the final biochip shown in Figure 4c. In a biochip for measuring monolayer cell cultures, the chip can be fully sealed during/after fabrication because later the cells can be loaded through the inlet as cell suspension [38]. However, this is no longer feasible when a tissue sample has to be measured. This is why our biochip design must have the capability to easily be assembled and disassembled for tissue mounting/removal. Since PDMS is biocompatible and permeable to gas [39], the viability of the tissue specimen is expected to be preserved on-chip with sufficient perfusion media. Indeed, subsequent detailed tests (not presented here) indicated that under certain conditions the viability of the tissue can be maintained for a period of at least 5 to 7 days. We believe that this is important, and thus our results reported here are extremely relevant, because it was reported that culturing samples in microfluidic devices provides a more holistic model as compared to those statically cultured in vitro because the microfluidic device can provide a more reproducible environment which better mimics *in vivo* conditions [40].

The TEER measuring setup is shown in Figure 4d. During measurement, the four electrodes are connected to the EVOM2 through a homemade connection adaptor.

### 3.4. Continuous On-Chip TEER Monitoring

**Figure 5:**
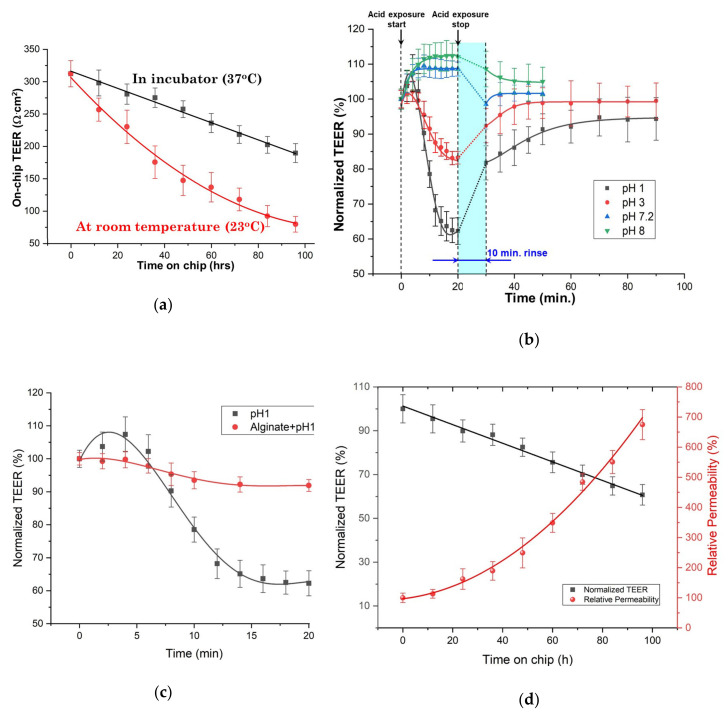
Data points for TEER measurements of tissues maintained in biochips in incubator and at room temperature, respectively. (**a**) On-chip TEER measurements of tissue viability (*n* ≥ 5; *R*^2^ = 0.991). (**b**) Normalized on-chip TEER responses of tissues exposed to media of different pH values (*n* ≥ 3). (**c**) On-chip TEER responses of tissues exposed to acidic solution with pH 1, with or without sodium alginate treatment tissues prior to acid exposure (*n* ≥ 3). (**d**) Normalized on-chip TEER (*n* = 4; *R*^2^ = 0.991) and relative permeability vs. duration on-chip (*n* = 4). (The numerical values for the data shown in Figure 5a,d can be found in Table S11, while those of Figure 5b,c can be found in Table S12, respectively, in the Supplementary Materials).

To demonstrate the ability of our tissue biochip to perform reliable continuous TEER monitoring, 6 mm disk specimens were maintained in 6 biochips with perfusion of culture media driven by a peristaltic pump at a flow rate of 0.6 mL/h. The biochips were kept in an incubator at 37 °C. For comparison, three biochips were kept instead at room temperature (23 °C). The TEER of each chip was measured at 52 h intervals and the obtained results are plotted in Figure 5a (The parameters of the fitting equations for the data shown in Figure 5a can be found in Tables S1 and S2 in the Supplementary Materials). It can be seen that the TEER values decreased in time due to the inherent reduction in the viability of the tissue with the passage of time, which intrinsically affected its integrity. The TEER value of fresh specimens is 312.3  ±  20.2 Ω·cm^2^, which is consistent with the values previously measured macroscopically with the “sandwich ring” set-up. However, by the end of the fourth day of on-chip culturing in the incubator the tissue’s TEER dropped about 40% to 189.7  ±  14.7 Ω·cm^2^. In contrast, the TEER of the tissues kept at room temperature had a much more rapid decrease. As it can be seen from Figure 5a, the TEER values of the samples maintained at room temperature dropped about 75% to 79.8  ±  12.1 Ω·cm^2^, indicating that sing the right (body-like) temperature is crucial for preserving the viability of tissue for longer periods.

We also performed the same acid exposure test which had been carried out using the “sandwich ring” method. For this purpose, the input of the peristaltic pump was connected to an acidified medium reservoir to supply low pH media to the on-chip specimens. The flow rate was set at 6 mL/h for 2 min. and then lowered to 1.2 mL/h. TEER readings were recorded at 2 min. intervals. After 20 min., the circulated fluid was changed back to normal medium in order to rinse the specimen at 6 mL/h for 10 min. For comparison, we also investigated the effects of solutions with other pH values, namely PBS solution at pH 7.2 and medium supplemented with sodium bicarbonate to up to pH 8, respectively. The recorded TEER values normalized as a percentage of the initial value before acid exposure are plotted in Figure 5b (The parameters of the fitting equations for the data shown in Figure 5b can be found in Tables S3–S6 in the Supplementary Materials). Surprisingly, the TEER values increased slightly in the first few minutes after acid exposure before starting to drop. The reason for the increase is not clear yet, but similar observations were reported previously by researchers working on human endoscopic esophageal biopsies [35]. At the end of the acid exposure period, the TEER reduced with 37.7% and 16.8% for the samples exposed to the pH 1 and pH 3 solutions, respectively. The recovery of the tissues was also continuously monitored after rinsing and the results show that the pH 3 exposed tissues almost fully recovered, but that was not the case for the pH 1 treated tissues, in agreement with the results obtained using “sandwich ring” setup.

At the end of the exposure to basic solutions, the TEER values increased with 8.6% and 12.1% for the samples exposed to the pH 7.2 and pH 8 solutions, respectively. After rinsing, the pH 7.2 exposed tissues almost fully recovered while the TEER was slightly increased, at 105%, in the case of pH 8 exposed tissue.

In another exposure test performed prior to the acid exposure, PBS with 2% sodium alginate was first supplied to the specimens for 20 min. followed by rinsing with normal media. The TEER responses of sodium alginate treated tissues to acid exposure are plotted in Figure 5c (The parameters of the fitting equations for the data shown in Figure 5c can be found in Table S7 in the Supplementary Materials). The obtained results show that alginate treated tissues became more resistant to acid exposure, indicating that the treatment offers good protection against the effects of such a treatment. This is because the alginate molecules can adhere on the surface of the epithelial tissue to form a protective layer [41].

We believe that all these experimental results clearly demonstrate that our biochip is an effective and useful substitute to an Ussing chamber that can be used in numerous practical tests of clinical relevance.

### 3.5. Relationship between Measured TEER and Tissue Permeability

TEER is considered to be a good indicator of barrier integrity of epithelial tissue samples, which directly determines the tissue permeability to various biomolecules. To quantify the tissue permeability, FITC-dextran was used as a flux tracer and its fluorescence intensity measured using the procedure outlined in Section 2.5. For the permeability test, effluents from the lower channer were collected at 12 h intervals and their fluorescence intensity were measured. The relative permeability *RP* was subsequently calculated as:

(1) $$RP=\frac{FI\left(t\right)-FI_{bckrnd}}{FI\left(t=0\right)-FI_{bckrnd}}\times 100,\;\mathrm{in}\;\left(\%\right)$$

where *FI*(*t*) is the fluorescence intensity at time *t* (in [h]), *FI*(*t* = 0) is the fluorescence intensity at time *t* = 0 (at the very beginning of the experiments), and *FI _bckgrnd_* is the background fluorescence intensity, which was measured after inserting an impermeable polytetrafluoroethylene (PTFE) film between the top and bottom perfusion channels in order to completely isolate them from one another. The variation in time of the resulting *RP* is plotted in Figure 5d together with that of the normalized on-chip TEER values (The parameters of the fitting equations for the data shown in Figure 5d can be found in Tables S8 and S9 in the Supplementary Materials). It can be seen that the data shown in Figure 5d clearly indicate that the decrease in TEER caused by exposure to acidic solutions is accompanied by a corresponding increase in permeability. A similar correlation was also mentioned in the literature when data from GERD patients had been analyzed [28], although in that case only two values were compared, namely from patients with GERD versus those from subjects with a healthy esophagus (controls). In contrast, Figure 5d presents a much more detailed picture, with many more data points which can thus allow a better correlation of TEER with permeability for a larger range of variation.

The previously mentioned paper of Takezono et al. [35] also measured more data points for both TEER and permeability of monolayers of cultured rat gastric epithelial cells (RGM-1) exposed to solutions of various pH values (3–7.4) for 60 to 120 min. However, permeability was measured differently, using the diffusion rate of ^14^C-labeled mannitol. Although in our case we compared permeability and TEER variations in time while Takezono et al. investigated the effects of exposure to media of different pH values, their data show similar variations with ours. Thus, permeability gradually increased over time for the samples exposed to the control medium (pH 7.4), just as in our case. For samples exposed to an acidic solution (pH 3), the gradual decrease of TEER was accompanied by an initial arrest of the ^14^C-labeled mannitol permeability for the first 20 min., after which it gradually increased, reaching the same level as that in controls in 60 min. [35].

### 3.6. Relationship between TEER and Tissue Viability

As shown previously in Figure 5a, the TEER of specimens maintained in the biochips decreases in time. We believed that this decrease in barrier integrity was due to the gradual reduction of tissue viability. The data shown in Figure 5d, which indicate that the relative permeability of the tissue did indeed rapidly increase in time while TEER decreased, further validated this assumption. However, as it can be seen from the curves in Figure 5b, while the TEER variation is linear, the permeability varies non-linearly (although it is a rather “weak” one, as indicated by the small value of the coefficient for *x*^2^, as it can be seen in the data of the fitting equations in the Supplementary Materials file). This may indicate that a more complex electrical model of the tissue, which would also take into consideration other components besides TEER, should be adopted. This can be achieved if, e.g., impedance measurements are performed instead of TEER, which can provide many more data about the analyzed tissue and its response to external changes [42]. Nevertheless, impedance measurements require a much more complex setup, a careful design of the experimental approach, as well as a more intricate extraction of the relevant parameters from the measured data which typically is done using proprietary software. Additionally, the careful interpretation of the measured data is one of the most difficult problems in the application of impedance measurement as sometimes component values which have no physical meaning may result. Therefore, TEER offers the advantages of simplicity and speed for a quick understanding of the first-order phenomena underlying the changes caused by external factors.

**Figure 6:**
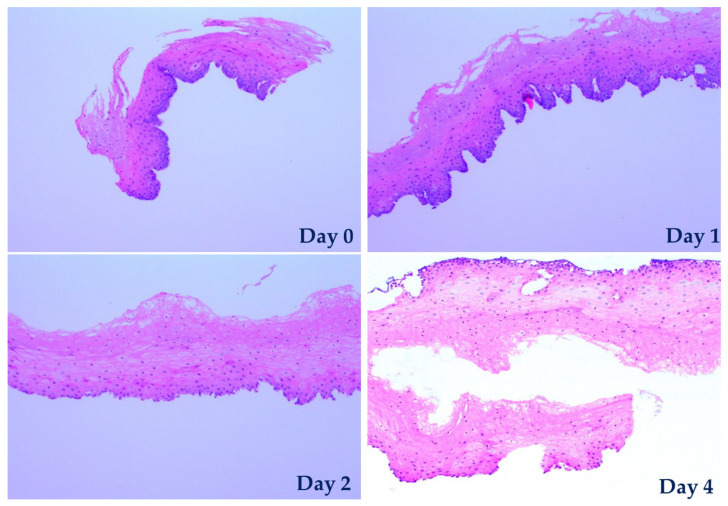
Images of H&E stained tissue specimens in day 0 (before placement in the chip) and after being cultured on-chip for 1, 2 and 4 days.

To fully confirm and verify that the permeability of the tissue did indeed rapidly increase in time, histological analyses were carried out. Histology analysis is considered the most reliable method to assess the viability of tissue to date. At culturing day 1, 2, 3 and 4, specimens cultured on-chip were taken out, fixed, and cut into thin slices 5 µm thick, followed by hematoxylin and eosin (H&E) staining. After staining, the viability of the specimens was determined qualitatively by observing the tissue morphology using an inverted microscope. The histology images are shown in Figure 6. Hematoxylin stains nuclei of cells in dark blue/purple, while eosin colors cytoplasm structures in various shades of red and pink. The observations from these histological images are in good agreement with our measured TEER values. On day 0 (the day when the epithelial tissue is extracted from the biopsied sample, processed, divided into pieces, and inserted in biochips), the tissue sample has the highest TEER value with excellent viability and distinguishable keratinocyte nuclei. The sample on day 1 is similar to that on day 0. On day 2 and day 4, however, the TEER drops by about 15% and 40%, respectively, with corresponding viability reduction of the upper keratinocytes by about 10% and 30% (as estimated approximately by a pathologist), displaying nuclear pyknosis and loss.

## 4. Conclusions

In this article, we demonstrated a “sandwich ring” method for faster and easier measuring the TEER of tissue specimens without using a Ussing chamber. The same concept was then applied to design and develop a dual-function tissue biochip for both temporary maintenance of tissue specimens as well as their continuous TEER monitoring. All the experimental results obtained using our tissue biochip clearly demonstrated that it is an effective substitute to an Ussing chamber. Moreover, we believe that our obtained results prove that this novel tissue biochip is much more practical, convenient and easier to use than an Ussing chamber due to its unique advantageous features, such as easy installation of the sample, re-usability, low-cost and rapid prototyping, small size, and enabling both long(er) term tissue viability and real-time monitoring of TEER.

With the aid of this biochip, porcine esophageal tissues were maintained viable and their TEER was monitored for up to four days. Due to the biochip’s microfluidics which enables significant flexibility in its application for various purposes, tests in which tissues were exposed to media to various pH values were easily performed and in conjunction with a permeability test they effectively demonstrated that such a tissue biochip can be used in a large range of possible applications which can subject the sample to various biochemical treatments. Our experimental results obtained from both the exposure of tissues to media of various pH values and the permeability tests led to two crucial conclusions. Firstly, and most importantly, our results demonstrate a clear strong correlation between the measured on-chip TEER and the tissue permeability (the former decreases when latter increases) which was confirmed by histopathological observations. The current state-of-art methods for assessing tissue viability are all involving biochemical treatments of the samples to generate optical contrast or detectable signals such as fluorescence for observation under microscope. Hence, these assessing methods are usually costly, time-consuming, labor intensive and more significantly, destructive to the samples. Secondly, the reliable correlation found between the TEER values and tissue viability demonstrates that TEER is an excellent alternative method for assessing tissue viability, as it is easier, faster, label-free, and non-destructive. These findings suggest that our biochip may find important clinical applications that could e.g., determine the most efficient drugs or assess the efficiency of a given treatment.

## Supplementary Materials

The following are available online at https://www.mdpi.com/1996-1944/13/10/2354/s1. The measured data which have been shown in the paper’s figures, and the parameters of the fitting equations are all listed in the Supplementary Materials file. Table S1: The fitting equation parameters obtained for the linear fit of the data shown in Figure 5a for tissues maintained in incubator at 37 °C. Table S2: The equation parameters obtained for the parabolic fit of the data shown in Figure 5a for tissues maintained at room temperature (23 °C). Table S3: The fitting equation parameters for the data corresponding to the TEER variations during exposure to acidic media shown in Figure 5b, fitted using a 4th order polynomial. Table S4: The fitting equation parameters for the data corresponding to the TEER variations during exposure to basic media shown in Figure 5b. Table S5: The fitting equation parameters for the data corresponding to the TEER variations after exposure to acidic media shown in Figure 5b, fitted using a log normal function. Table S6: The fitting equation parameters for the data corresponding to the TEER variations after exposure to basic media shown in Figure 5b. Table S7: The fitting equation parameters for the data corresponding to the variations of on-chip TEER responses of tissues exposed to acidic solution with pH 1, with or without sodium alginate treatment, shown in Figure 5c, fitted using a 4th order polynomial. Table S8: The fitting equation parameters obtained for the linear fit of the normalized TEER variation shown in Figure 5d. Table S9: The fitting equation parameters obtained for the parabolic fit of the relative permeability data shown in Figure 5d. Table S10: The numerical values for the data plotted in Figure 3a,b. Table S11: The numerical values for the data plotted in Figure 5a,d. Table S12: The numerical values for the data plotted in Figure 5b,c.
