# Supplementary material for: Low-Cost Method and Biochip for Measuring the Trans-Epithelial Electrical Resistance (TEER) of Esophageal Epithelium
Source: Materials (Basel). 2020 May 20;13(10):2354. doi: 10.3390/ma13102354 (PMC7288155; doi:10.3390/ma13102354)
Supplement: Supplementary file 1 [file materials-13-02354-s001.pdf]

materials

MDPI

Supplementary Materials

Low-Cost Method and Biochip for Measuring the Trans-Epithelial Electrical Resistance (TEER) of Esophageal Epithelium

Daniel Puiu Poenar [1,*], Guang Yang [1], Wei Keat Wan [2] and Shilun Feng [1]

[1] VALENS-Centre for Bio Devices and Signal Analysis, EEE School, Nanyang Technological University, Blk S1, Level B4, Unit 06 (S1-B4b-06), 50 Nanyang Avenue, Singapore 639798, Singapore; YANG0389@e.ntu.edu.sg (G.Y.); shilun.feng@ntu.edu.sg (S.F.)

[2] Histopathology Department, Singapore General Hospital (SGH), 20 College Road, Academia, Level 10, Diagnostic Tower, Singapore 169856, Singapore; wan.wei.keat@singhealth.com.sg

* Correspondence: epdpuiu@ntu.edu.sg; Tel.: +65-6790-4237

Received: 18 April 2020; Accepted: 18 May 2020; Published: date

1) Best fitting curves

We hereby attach the values of all the coefficients for all the fitting curves extracted (using ORIGIN) for the data points plotted in all the paper's Figures.

Table S1. The fitting equation parameters obtained for the linear fit of the data shown in Figure 5a for tissues maintained in incubator at 37 °C.

Equation	$a_0 + a_1x$
Weight	Instrumental
Intercept a_0	316.349 ± 2.943
Slope a_1	–1.327 ± 0.048
Residual sum of squares	0.517
Pearson's r	–0.995
R-square (COD)	0.991
Adj. R-square	0.99

Table S2. The equation parameters obtained for the parabolic fit of the data shown in Figure 5a for tissues maintained at room temperature (23 °C).

Equation	$a_0 + a_1x + a_2x^2$
Weight	Instrumental
Intercept a_0	306.821 ± 6.33
a_1	–3.921 ± 0.307
a_2	0.0164 ± 0.003
Reduced χ_S	0.159
R-square (COD)	0.994
Adj. R-square	0.992

Figure 5b shows the variation of TEER during and after exposure to solutions with various values. Hence, in each case there are 2 sets of data, one for the behaviour while being exposed to the solution, and the other for the recovery after the 10 min. rinse. Because the behaviour was very different in the two cases, each data set was fitted with a different function, detailed in Tables S3 to S6 below.

Table S3. The fitting equation parameters for the data corresponding to the TEER variations during exposure to acidic media shown in Figure 5b, fitted using a 4th order polynomial.

**Equation**	$y = a_0 + a_1x + a_2x^2 + a_3x^3 + a_4x^4$	
**Sample/Exposure to acidic solution with:**	**pH 1**	**pH 3**
a_0	99.419 ± 1.439	99.70085 ± 0.67147
a_1	7.339 ± 1.561	2.42938 ± 0.52609
a_2	–1.806 ± 0.361	–0.60408 ± 0.11687
a_3	0.106 ± 0.028	0.03432 ± 0.00892
a_4	–0.002 ± 6.94×10⁻⁴	–6.12821×10⁻⁴ ± 2.18×10⁻⁴
Reduced χ_S	0.316	0.078
R-square (COD)	0.991	0.996
Adj. R-square	0.986	0.993

Table S4. The fitting equation parameters for the data corresponding to the TEER variations during exposure to basic media shown in Figure 5b.

**Equation**	$y = A_2 + \frac{A_1 - A_2}{1 + \left(\frac{x}{x_0}\right)^p}$	
**Sample/Exposure to basic solution with:**	**pH 7.2**	**pH 8**
A_1	100.016 ± 0.422	100.046 ± 0.345
A_2	108.804 ± 0.155	113.078 ± 0.478
x_0	1.995 ± 0.14	3.065 ± 0.258
p	2.905 ± 0.637	1.732 ± 0.255
Reduced χ_S	0.021	0.014
R-square (COD)	0.985	0.994
Adj. R-square	0.979	0.991

The second set of data shown in Figure 5b, for the variation corresponding to the recovery that followed after exposure to the media of various pH values and its subsequent 10 min. rinse, have their parameters detailed in Tables S5 and S6.

Table S5. The fitting equation parameters for the data corresponding to the TEER variations after exposure to acidic media shown in Figure 5b, fitted using a log normal function.

**Equation**	$y = y_0 + \frac{A}{W \cdot x \cdot \sqrt{2\pi}} \exp\left[-\frac{\left(\ln \frac{x}{x_C}\right)^2}{2W^2} \right]$	
**Sample/Exposure to acidic solution with:**	**pH 1**	**pH 3**
y_0	94.799 ± 0.809	99.295 ± 0.119
x_C	31.943 ± 2.306	29.087 ± 1.558
W	0.401 ± 0.105	0.195 ± 0.038
A	–392.617 ± 28.144	–101.722 ± 6.678
Reduced χ_S	0.0227	0.003
R-square (COD)	0.983	0.993
Adj. R-square	0.973	0.989

Table S6. The fitting equation parameters for the data corresponding to the TEER variations after exposure to basic media shown in Figure 5b.

Equation	$y = y_0 + A_1\exp(-x/t_1)$	
**Sample/Exposure to basic solution with:**	**pH 7.2**	**pH 8**
y_0	101.723 ± 0.075	104.87422 ± 0.20607
A_1	$-4.32\times10^{7} \pm 2.512\times10^{8}$	2675.391 ± 3655.743
t_1	1.825 ± 0.645	4.585 ± 0.971
Reduced χ_S	0.0028	0.0027
R-square (COD)	0.971	0.987
Adj. R-square	0.941	0.975

The parameters for the fitting equations of the curves shown in the other Figures of the paper are also given in the following Tables.

Table S7. The fitting equation parameters for the data corresponding to the variations of on-chip TEER responses of tissues exposed to acidic solution with pH 1, with or without sodium alginate treatment, shown in Figure 5c, fitted using a 4th order polynomial.

Equation	$y = a_0 + a_1x + a_2x^2 + a_3x^3 + a_4x^4$	
**Sample/Exposure to acidic solution with:**	**pH 1**	**Alginate + pH 1**
a_0	99.419 ± 1.439	99.79979 ± 0.5936
a_1	7.339 ± 1.561	0.60884 ± 0.60736
a_2	−1.806 ± 0.361	−0.25623 ± 0.1571
a_3	0.106 ± 0.028	0.01716 ± 0.01324
a_4	$-0.002 \pm 6.94\times10^{-4}$	$-3.43\times10^{-4} \pm 3.44\times10^{-4}$
Reduced χ_S	0.316	0.10137
R-square (COD)	0.991	0.983
Adj. R-square	0.986	0.961

Table S8. The fitting equation parameters obtained for the linear fit of the normalized TEER variation shown in Figure 5d.

Equation	$a_0 + a_1x$
Weight	Instrumental
Intercept a_0	101.286 ± 0.943
Slope a_1	−0.425 ± 0.015
Residual sum of squares	0.517
Pearson's r	−0.995
R-square (COD)	0.991
Adj. R-square	0.99

Table S9. The fitting equation parameters obtained for the parabolic fit of the relative permeability data shown in Figure 5d.

Equation	$a_0 + a_1x + a_2x^2$
Weight	Instrumental
Intercept a_0	96.873 ± 8.509
a_1	0.963 ± 0.663
a_2	0.055 ± 0.008
Residual sum of squares	2.348
R-square (COD)	0.994
Adj. R-square	0.992

2) Data of all the other plots

Table S10. The numerical values for the data plotted in Figure 3a,b.

Section of thoracic esophagus	TEER (Ω · cm²)	Standard deviation (S.D.)	Normalized TEER (as % of value before exposure)	pH 1	S.D.	pH 3	S.D.	pH 7.2	S.D.
upper	289.2	16.45	Before acid exposure	100	7.73	100	6.69	100	7.7
middle	310.5	24.01	After acid exposure	66.5	5.45	87.4	4.48	108.1	4.5
lower	306.8	20.53	1 hour after rinse	91.8	6.26	99.6	4.08	102.2	4.2

Table S11. The numerical values for the data plotted in Figure 5a,d.

Time on chip (h)	TEER (Ω · cm²) @37°C	S.D.	Normalized TEER (as % of value before exposure)	S.D.	Relative Permeability (as % of value at t=0)	S.D.	TEER (Ω · cm²) @23°C	S.D.
0	312.3	20.17	100	6.46	100	15.7	312.3	20.2
12	298.0	19.95	95.4	6.39	113	14.6	256.8	17.8
24	280.8	15.69	89.9	5.02	162	34.1	230.3	25.7
36	275.3	15.14	88.1	4.85	189	30.9	175.6	25.2
48	257.6	13.02	82.5	4.17	249	49.8	147.4	23.4
60	236.1	14.81	75.6	4.74	349	31.6	136.9	22.8
72	218.5	13.66	69.9	4.37	485	27.7	118.1	17.5
84	202.5	12.97	64.8	4.15	551	38.4	92.3	16.3
96	189.7	14.69	60.7	4.70	675	50.1	79.7	12.1

Table S12. The numerical values for the data plotted in Figure 5b,c.

Acid exposure (min)	pH 1		pH 3		pH 7.2		pH 8		Alginate+pH1	
	Normalized TEER (as % of value at *t*=0)	S.D.	Normalized TEER (as % of value at *t*=0)	S.D.	Normalized TEER (as % of value at *t*=0)	S.D.	Normalized TEER (as % of value at *t*=0)	S.D.	Normalized TEER (as % of value at *t*=0)	S.D.
0	100	2.6	100	2.5	100	2.9	100	2.9	100	1.9
2	103.7	4.4	101.5	2.7	104.5	2.7	104.6	3.7	99.3	2.3
4	107.4	5.3	102.2	2.9	107.4	2.5	107.1	4.5	99.8	2.5
6	102.2	5.1	99.6	2.2	108.9	2.8	109.9	4.4	97.9	2.2
8	90.3	4.9	95.5	3.9	109.5	3.1	111.5	4.2	95.3	3.4
10	78.5	3.8	91.5	3.4	108.9	2.7	111.9	3.7	93.5	2.6
12	68.2	4.5	87.5	2.8	108.8	2.8	112.3	3.8	--	--
14	65.1	4.1	86.2	2.5	108.6	2.7	112.1	4.1	92.2	2.3
16	63.6	4.2	85.1	2.4	108.6	2.4	112.4	4.4	--	--
18	62.5	3.5	83.1	1.9	108.7	2.2	112.4	3.6	--	--
20	62.3	3.8	83.2	1.8	108.6	2.0	112.1	3.9	91.9	1.8
Rinse	--	--	--	--	--	--	--	--		
30	81.818	4.9	92.461	4.9	98.6	7.1	108.7	5.1		
35	84.461	5.3	95.497	5.3	101.5	3.3	106.3	4.5		
40	86.152	5.1	97.906	5.1	101.8	2.3	105.2	3.7		
45	88.372	3.8	99.058	4.7	101.8	2.5	104.8	5.3		
50	91.438	4.4	98.953	4.4	101.6	2.2	105.1	4.1		
60	92.178	4.7	98.848	6.5						
70	94.82	5.3	99.372	5.3						
80	94.195	6.4	99.372	4.4						
90	94.383	6.1	99.581	5.1						

© 2020 by the authors. Submitted for possible open access publication under the terms and conditions of the Creative Commons Attribution (CC BY) license (http://creativecommons.org/licenses/by/4.0/).
